# Examining Participant Adherence with Wearables in an In-the-Wild Setting

**DOI:** 10.3390/s23146479

**Published:** 2023-07-18

**Authors:** Hannah R. Nolasco, Andrew Vargo, Niklas Bohley, Christian Brinkhaus, Koichi Kise

**Affiliations:** 1 Graduate School of Informatics, Osaka Prefecture University, Sakai 599-8531, Japan; df104001@st.osakafu-u.ac.jp; 2 Department of Core Informatics, Graduate School of Informatics, Osaka Metropolitan University, Sakai 599-8531, Japan; kise@omu.ac.jp; 3 Department of Computer Science, University of Kaiserslautern-Landau, 67663 Kaiserslautern, Germany; nbohley@rhrk.uni-kl.de (N.B.); brinkhau@rhrk.uni-kl.de (C.B.)

**Keywords:** wearable devices, in-the-wild, self-tracking

## Abstract

Wearable devices offer a wealth of data for ubiquitous computing researchers. For instance, sleep data from a wearable could be used to identify an individual’s harmful habits. Recently, devices which are unobtrusive in size, setup, and maintenance are becoming commercially available. However, most data validation for these devices come from brief, short-term laboratory studies or experiments which have unrepresentative samples that are also inaccessible to most researchers. For wearables research conducted in-the-wild, the prospect of running a study has the risk of financial costs and failure. Thus, when researchers conduct in-the-wild studies, the majority of participants tend to be university students. In this paper, we present a month-long in-the-wild study with 31 Japanese adults who wore a sleep tracking device called the Oura ring. The high device usage results found in this study can be used to inform the design and deployment of longer-term mid-size in-the-wild studies.

## 1. Introduction

Representative samples are uncommon when it comes to in-the-wild studies employing wearable technologies. Even more infrequent seem to be experiments conducted over an extended period of time. This comes as no surprise given how expensive mid-size experiments employing wearables can be: wearables and sensors may be affordable at an individual level but can become costly when purchased in bulk. This is potentially why many studies place a particular focus on creating and implementing low-cost sensors [1,2]. In addition to this, paying participants over an extended period of time can be an expensive commitment and is risky when dealing with in-the-wild setups. Participants may end up dropping the study prematurely or use the wearable incorrectly if they are left unsupervised or unguided, preventing researchers from producing viable results from their experiment. Since studies that are dropped are not published, it is hard to know how often this happens.

Researchers usually accept the most readily available participants due to limitations in available populations, which means that they often recruit university students in their experiments as well as mostly male participants [3]. The former is due to proximity, while the latter is due to men being typically more willing to share personal data and become early adopters of wearable technologies [3]. This may explain why males tend to make up a higher percentage of volunteers in experiment samples despite genuine attempts to recruit an equal number of women. While it is reasonable to seek out study participants through convenient methods, these can come with unintended consequences. For example, working with students may lead them to feel a pressure to participate or to provide the researcher with favorable results simply because of their position of authority, which introduces a complicated power dynamic into the study [4]. Moreover, working with primarily male and/or university-aged participants leaves us with a gap in understanding of how other members of society may realistically build an affinity to a wearable device.

In this article, we present results from a 30-day in-the-wild study that made use of a device called the Oura ring, a physiological sensing device specializing in tracking sleep. An in-the-wild study, in the context of ubiquitous computing, is one in which participants in an experiment interact with a device in their natural environment, and interaction and control from the investigators is minimized. There is a spectrum of in-the-wild studies, including those with many interruptions and prompts from the investigators [5] and those with few interaction. In this study, participants were not being actively or constantly monitored and they were only prompted once a week if it appeared that they were not using the ring. The participants also did not have to adhere to any schedule or series of tasks and were allowed to integrate the ring into their daily lives however they pleased in the 30 days of the experiment.

In this paper, we seek to answer the following research questions:

- **RQ1:***Can a satisfactory number of participants from the sample successfully wear the Oura ring consistently in a long-term in-the-wild study?*—We consider a participation rate close to or above 80% to be an acceptable rate as it indicates active usage in four out of every five days in the experiment period. This is important because it means we are unlikely to have multiple days of missing data and it demonstrates consistent usage.
- **RQ2:***Are there differences between broad-based demographics in terms of usage trends and compliance with wearing the ring?*
- **RQ3:***Can confusion over wearing the Oura ring (e.g., interpretation of the data, interaction with the application, difficulties syncing/charging the device) impact compliance with wearing the device?*

## 2. In-the-Wild Studies

There is still a need for more in-the-wild studies with representative populations that properly assess user interactions in real-life settings [6]. Although some in-the-wild experiments on wearables already exist, they possess other design limitations. For example, Alharbi et al.’s study on the authenticity of captured behavior using a wearable camera in-the-wild confronts the psychological effects of the surveillance properties of certain devices, but the study was only run for a brief duration and still included structured activities. Some participants of the study also did not take the device home with them [7]. In another in-the-wild study focused on eye-motion tracking, the participant pool was made up of just 16 individuals and incorporated an obtrusive notification system which activated every two hours [5]. Other studies suffer from an issue of imbalanced demographics, in which most subjects are young and/or identify as male [8,9] or the subjects are all university students [10,11]. There is one experiment that was conducted in-the-wild with the Fitbit that ran with a decently sized generalized sample over an extended period of time. However, they do not discuss granular adherence to the device at an individual level and only provide aggregate information on how often the Fitbit was worn [12].

Studies on readily available people can easily be seen as a “safe option”, but there is value in going outside of that target to expand our understanding of how people interact with devices. For manufacturers of wearable technologies, there is a possibility that they are failing to maximize the potential of their wearables because the devices do not apply to everyone. This is just as much of a concern for designers and researchers. One may perform targeted studies which focus on underrepresented populations (e.g., women, the elderly, people with disabilities) to discover more information about these subgroups, but a study covering the general population can obtain this information and a lot more in just one experiment. Limitations that we would have otherwise not found through a narrower sample may be made much more apparent through the lens of a broader population. One example of this is in considering the unique circumstances of a pregnant woman when measuring her weight [13].

A reasonable solution to ensure compliance and to balance drawbacks expected from in-the-wild studies is to perform a laboratory study. However, regardless of data quality, laboratory conditions cannot account for the variety of dynamic contexts in daily life that influence the capture of user behavior [14]. When applied to data acquired from free-living populations, laboratory-calculated algorithms are 13% less accurate [15]. While lab experiments have the benefit of minimizing confounding variables, they alone cannot depict a precise picture of real world conditions [16]. Apart from these issues, laboratory studies can also be very costly themselves if conducted over an extended duration. Paying for participants to come to a laboratory setting for a month would be expensive, and there are limitations to what is possible given COVID-19 restrictions. Performing more studies with in-the-wild setups can be more affordable while allowing researchers to achieve a more authentic picture of how people interact and engage with wearables in their natural environment. To the best of our knowledge, our study is novel in the aspect that we focus on continuous wear time of a wearable and the analysis of user engagement over the course of an experiment timeline.

## 3. The Oura Ring

### 3.1. Overview

The Oura ring is a commercially available consumer-oriented wearable device fitted with research-grade sensors. The parameters measured by the device range from degrees of physical activity and respiration to heart rate variability (HRV) and body temperature. All captured data are displayed on the device’s accompanying mobile application. Although comprehensive in its tracking of physiological data, the ring specializes in monitoring sleep: It can identify the differences between sleep stages and detect the duration of light, deep, and rapid-eye movement (REM) sleep. This information, along with its assessment of the user’s key body signals, allows the ring to reliably report one’s readiness or physical preparedness everyday. Constant and extended wear of the ring is essential for it to draw up a blueprint of the wearer’s normal vitals—this allows it to accurately detect even the most minute changes in the body and gives it the ability to detect fluctuations in stress and indications of recovery.

Laboratory studies have confirmed the accuracy of the Oura ring’s sleep stage detection and prediction of sleep quality [17]. The nature of the Oura ring as a device worn on the finger rather than on the wrist adds to its reliability given that wrist-worn devices tend to be less consistently accurate [18]. The ring itself costs around USD 300 for a base model. Use of the Oura ring requires a monthly membership fee to access the application’s daily in-depth analysis, live heart rate monitoring, personalized health insights and recommendations, and temperature trend monitoring, which costs USD 5.99 per month. These are reasonable costs at an individual level but can add up quickly for any research laboratory.

The Oura ring is an attempt to use a consumer wearable device to recreate laboratory studies that employ more highly-developed sensors, such as Chyad et al.’s study on sleep apnea using biomedical sensors [19] and O’Hare et al.’s comparative analysis with no-contact radio-frequency sleep measurement devices [20]. Several papers have already been published that demonstrate the abilities of the Oura Ring, many of which have compared the accuracy of the ring to medical-grade sensors such as electrocardiography (ECG) [21] for heart rate monitoring and research actigraphy and polysomnography for sleep tracking [22]. Moreover, a number of partial in-the-wild studies have been conducted on early detection of COVID-19 [23] and longitudinal studies examining people’s experiences with the Oura ring as a sleep tracking device have also been coming to the fore [24].

Making use of a consumer wearable such as the Oura ring has several benefits for researchers. It can help cut down on costs and make the experiment setup much easier compared to laboratory studies. It is also worthwhile to run more experiments attempting to validate the Oura ring’s usability in-the-wild as having a consumer wearable that comes close to medical-grade equipment can potentially improve access to healthcare and create inexpensive options for outpatient monitoring. A possible negative, however, is the greater chances of wearable misuse in studies conducted in-the-wild. Since participants are not actively being guided through the experiment or given a script to work with, there is much less control for user error.

### 3.2. Using the Oura Ring

The Oura ring has sizes ranging from 16.45 mm in diameter (US size 6) to 22.2 mm (US size 13). Determining your size takes roughly a day to accomplish. Oura has a free sizing kit containing accurate samples of all 8 sizes: The user must choose a size to test out on the finger they intend to wear the ring on at all times (Oura recommends the index finger). A good fit is described as having the sensors pressing lightly against the underside of the finger. The ring should not be easy to twist. Since hands have the tendency to change slightly in size throughout the day, Oura recommends wearing the sample for one full day to ensure that it is consistently comfortable.

**Figure 1:**
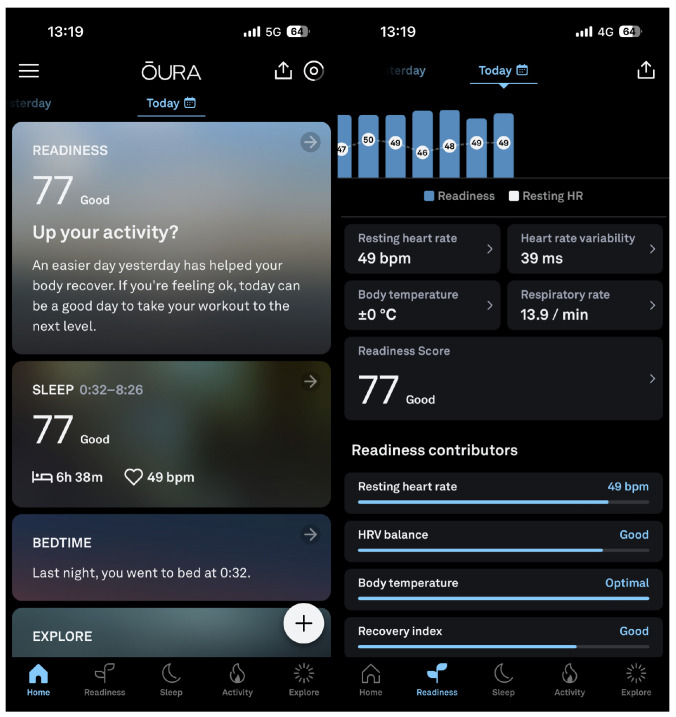
Oura’s app interface which shows the Readiness and Sleep Scores and the factors contributing to their calculation.

It takes two weeks for the Oura ring to build a comprehensive blueprint of the user’s physiology. This includes the user’s typical sleep rhythm, their normal heart rate, and their average body temperature (Figure 1). Once it has gathered enough information about its user, consistent adherence to wearing the ring is necessary for Oura to maintain its understanding of the wearer in order to report accurate readiness and sleep scores (Figure 1) (https://ouraring.com/blog/readiness-score/ (accessed on 15 March 2023)). The ring should be worn throughout the day with the exception of short tasks in which keeping the device on might be uncomfortable (e.g., weightlifting) (https://support.ouraring.com/hc/en-us/articles/4408961184147-General-FAQs (accessed on 15 March 2023)). Wearing the ring right before sleeping, all throughout the night, and in the daytime upon waking is also vital to ensure that the device captures the user’s sleep stages accurately. To sync their data, the user must access the Oura application on their mobile phone; the ring can store up to a week’s worth of data without syncing, so it is recommended that app syncing be performed on a regular basis. The ring is only charged when it alerts to having a low battery percentage (the ring’s battery lasts for several days, so this should happen every 4–7 days).

## 4. Materials and Methods

### 4.1. Experiment Background

As part of the Osaka Grand Challenge (https://www.ids.osaka-u.ac.jp/ildi/en/overview/research-projects/p10/index.html (accessed on 15 March 2023)), we were given the opportunity to recruit participants from the general population to take part in experiments with wearable devices. One important aspect of the Osaka Grand Challenge is to make pervasive computing technologies useful and beneficial for all members of society. This requires us to find ways to test and deliver technologies to a broader section of the population as compared to studies that recruit only from universities.

When preparing these studies, there was significant concern over how members of the general population would interact with the Oura Ring. As a nascent technology, wearables such as the Oura Ring might only be attractive for a sub-set of the population, making the ring look more usable in the real-world than it actually is. Thus, we focused this initial study on how the participants would interact with and wear the device.

Since participants were not being directly monitored or guided in their use of the ring, there were several risks and opportunities for this study:

- Participants are essentially on their own as a normal consumer and may find Oura’s app interface confusing, which may help us identify weak points in the application’s user experience.
- Participants may find their data difficult to parse or derive meaning from. If this is the case, it may help us in determining whether such an obstacle can affect their affinity to the device or their compliance with wearing it.
- Participants may find the ring cumbersome to wear daily and thus may put it on inconsistently or choose to drop the experiment early. This can provide us with more insight on what aspects of the ring influence their comfort and accessibility for daily use.
- Participants may fail to charge their ring or sync their data properly, which may indicate pitfalls in the clarity of the ring’s provided instructions.

### 4.2. Participants

From an initial pool of 32 participants, a cohort of 31 Japanese adults (F = 16, M = 15) between the ages of 20 and 50 (AVG = 33, SD = 9.09) were successfully initiated into the study through a recruitment company. The company put out a non-specific call for participating in scientific experiments and people who fit the criteria for our study were able to apply. We specifically requested the company to form a participant pool with a normal distribution in age range (Table 1) and a balanced distribution in terms of gender. We did not provide the company with any specific exclusion criteria, but participants were required to demonstrate some familiarity with the English language through the completion of a simple English questionnaire during the recruitment process. Participants were also able to opt into declaring any chronic health issues, but none of the subjects chose to disclose such information.

The participant who was excluded from the initial pool of 32 did not attend the orientation session or submit their signed consent form and thus had to be removed from the study. One user out of the thirty-one participants dropped the study after 9 days, leaving us with thirty users (F = 16, M = 14). Due to the small sample size, the age-range was limited so that age-effects could be observed with reasonable statistical power. We ran a Mann–Whitney U test to check whether there were significant age differences between the two genders and the results were insignificant. However, the Shapiro–Wilk Normality Test diagnosed that both age and weight were not normally distributed.

The researchers had no control or direct knowledge of who these individuals were. Remuneration was fixed at JPY 10,000 (approximately USD 75). Participants were not given any minimum requirements in order to qualify for compensation. The study received ethical approval in advance by the ethics committee of the Graduate School of Engineering of Osaka Metropolitan University (formerly Osaka Prefecture University).

### 4.3. Data Collection

We first provided participants with an Oura ring sizing kit during an in-person orientation administered by the last author of this paper, and allowed them to determine their appropriate ring size on their own with the directions provided by Oura. Once users received their Generation 3 ring, they were then asked to wear it for a minimum of 30 days. Two surveys were also administered to collect participant sentiments: The first was sent out in the middle of the experiment (15th day) and the second was released upon the conclusion of the experiment (31st day). Both surveys were disseminated through email and were taken on Qualtrics. Participants also completed two psychomotor vigilance tasks (PVT) on their personal phones each day, although this is not the focus of this article.

We had access to all of the physiological and situational data that the Generation 3 Oura ring tracks and registers into the cloud. If users logged their physical activity or health condition into the mobile application, this information was available to us. We could also view the user’s heart rate as measured by the ring every five minutes, their body temperature and respiration, their sedentary or inactive times in which they were not moving, and their total number of steps made during the day. In addition to this, we could see the precise sleep and wake times of each user, their total sleep duration, and the lengths of their light, deep, and REM sleep throughout the night. The most important data that were collected and analyzed for the purpose of this study was the non-wear time (NWT) of each user: we could retrieve the number of hours in which users did not wear the ring on each day of the experiment.

Users were reminded about the ring via email at two points during the 30 days if they were not syncing their ring data at all. The first email was especially important since it reminded some users to start wearing the ring. The initial email was sent to 7 participants, while the second email was sent to 5 participants. Instructions were not provided on how to best maximize the ring’s functionalities; instead, users were encouraged to engage with the technology however they deemed fit according to their lifestyle, even if it meant removal or low adherence to ring wearing. Users were also able to opt into wearing the ring past the 30 days, but were asked to return the ring before 40 days.

## 5. Results

In our analysis of the data, we took a look at each participant’s non-wear time (NWT) and their usage distribution in order to obtain a clear idea of their compliance with wearing the ring. NWT is a daily metric collected by the Oura ring that shows the number of hours that the user had their ring off. We visualized the differences in mean NWT between male and female subjects for each experiment day using a line graph and included the standard deviation to see the variances of all averages. We also compared the distribution of NWT between younger and older individuals in the sample by using a box plot to see whether a certain age bracket wore the ring more than the other. Included in the results is a heat map containing the daily average total wear-time of all participants per experiment day.

In order to capture a comprehensive image of compliance, we also looked at the number of active days that each participant had (i.e., the number of days in which the device was worn at all) to see how consistent users were at wearing the ring throughout the duration of the experiment. We combined these factors into a normalized metric to calculate an **adherence score** for each user before comparing the results to the survey responses. In this way, we could see how the impressions of the participants compared to their actual data. We then ran a generalized linear model (GLM) to determine whether any sociodemographic factors had a significant influence on the adherence score.

### 5.1. Examining Non-Wear Behavior

We examined the daily NWT of all users throughout the 30-day experiment period to determine the average compliance rate across all participants. We then observed whether there were any differences in compliance across age and gender.

**Figure 2:**
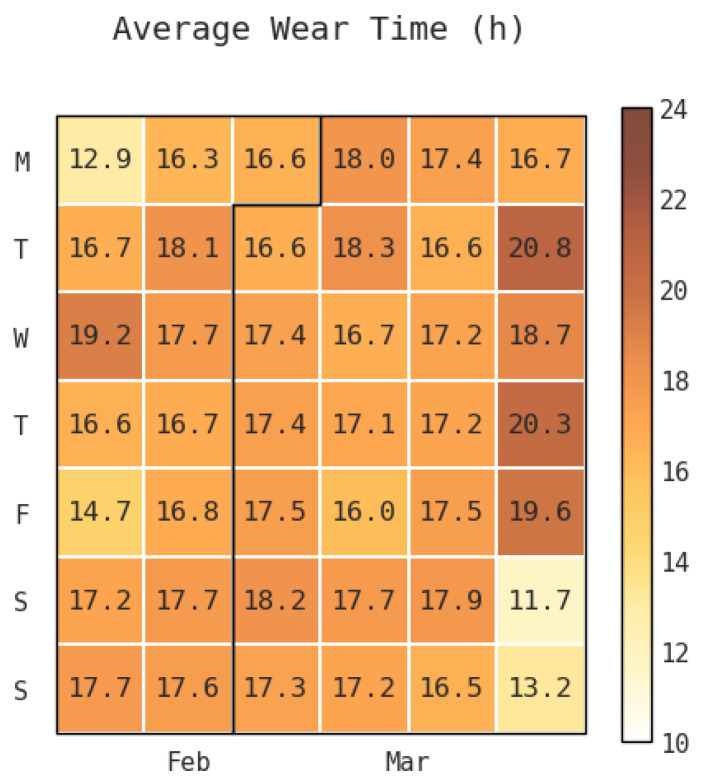
The daily average total wear-time across all participants per experiment day.

Starting from day 2 of the experiment, the daily average wear time across all participants did not fall below 12 h: Daily average wear times typically ranged between 15 and 19 h with very little variation (SD = 1.7) (Figure 2). The values did not deviate respective to the day of the week and remained relatively consistent throughout the 30-day experiment duration. However, the daily median wear time for all participants per experiment day was noticeably higher than the daily average: The values ranged from 18–21 with the exception of two days where the median was 16.3 and 17.6 (SD = 2.2). This indicates that most users have higher actual daily wear times and the average is being brought down by outliers.

**Figure 3:**
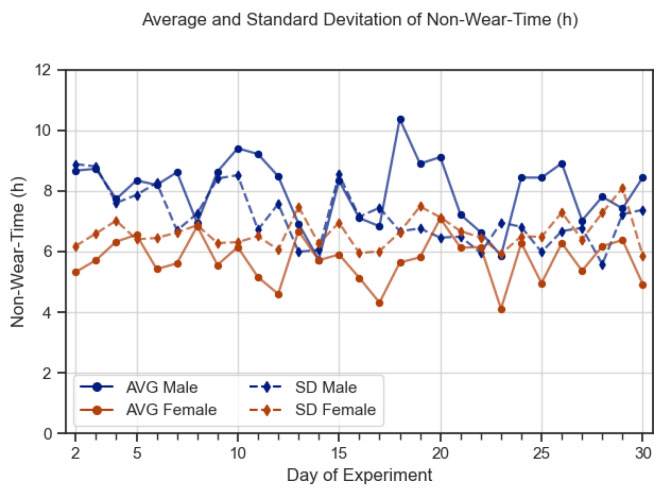
Difference in average NWT between male and female participants across experiment days.

**Figure 4:**
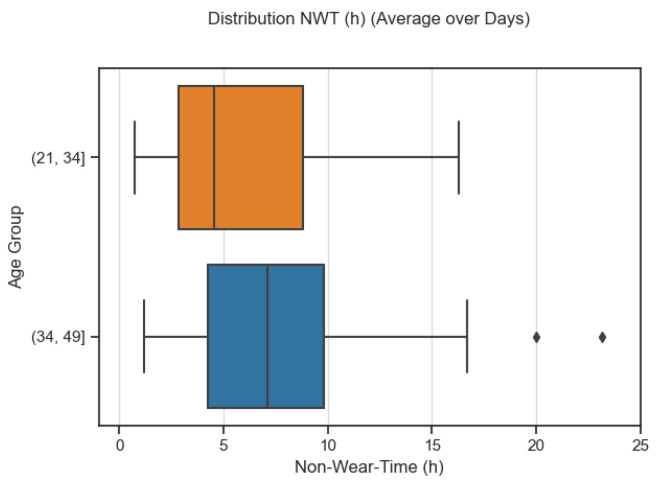
Distribution of NWT in younger and older age brackets.

Women appeared to wear their rings more consistently over longer periods compared to men: their average daily NWT was 5.64 h (SD = 6.32) while men averaged 7.75 h of NWT a day (SD = 6.96) (Figure 3). Meanwhile, younger participants (21–34) had a mean NWT of 5.39 (SD = 5.34) while older individuals (35–49) had a mean NWT of 8 (SD = 7.71). Despite this contrast between age brackets, the interquartile range between the two groups almost fully aligns, meaning the spread of values are almost the same for both groups (Figure 4).

### 5.2. Scoring Adherence

In order to properly assess whether the perceived differences in non-wear habits among users were significant, we devised a normalized metric that could consider both daily non-wear time and day-to-day consistency in wearing the ring. This allowed us to account for multiple combinations of possible adherence to the device and appropriately reward regularity. For example, some users with a high total wear-time (i.e., low total NWT) could have still gone multiple days in a row without wearing the ring, while other users with a lower total wear-time (i.e., high total NWT) may have never missed a day without putting it on. This metric takes each user’s total duration of wear-time between days 2 and 30 of their participation and combines it with the their total number of active days to obtain an **adherence score** out of 200: (1−(TotalWearTime/720)∗10+(TotalActiveDays/30)∗10). All days with less than 24 h of NWT count as active days.

Most users have a high NWT on the first day because they are putting on the ring for the first time and the ring has no data from the previous hours of the day. To avoid skewing the results, the total wear time calculation begins at day 2. If the user stopped wearing the ring before the 30th day, then their participation hours and number of participation days would also be adjusted (e.g., users who stopped on day 27 had their hours divided by 624 instead). Only four users did not complete all 30 days (one user = 23 days, two users = 27 days, one user = 29 days).

We divided each participant’s adherence score out of the maximum score of 200 to determine their participation rate, since the adherence score accounts for both their total number of days active during the experiment and the total number of hours in which they wore the ring. Twenty-four out of thirty-one users had a satisfactory participation rate equal to or above 80%, while two users fell slightly short at 78%. Only four users had low participation rates that were less than 75% (Table 2). This means that 87% of the participant pool (26 out of 30) successfully wore the ring at a consistent enough rate to provide data for analysis, which is greater than the percentage that we considered as satisfactory in our RQ1.

### 5.3. Statistical Analysis of Adherence Score

We analyzed the data with a Generalized Linear Model (GLM) using the Gamma distribution based on the distribution of the dependent variable. The initial GLM was fit with the adherence score as the response variable while age, weight, and gender were set as predictor variables. Since both age and weight were not normally distributed, we normalized the variables with a log transformation. We then conducted a step-wise elimination and removed the least significant variables, starting with weight and then age, until we were left with gender. The results were insignificant, meaning none of these sociodemographic factors have any impact on the adherence score.

### 5.4. Insights from Survey Results

We considered several confounding factors throughout the duration of the experiment: There was a possibility due to the lack of monitoring and guidance that users would find the application confusing or the data provided difficult to understand. We were also aware of the possibility that the participants would fail to charge the ring, forget to sync it to their cloud, or find it inconvenient to use regularly, leading to a premature departure from the study or to us receiving insufficient data. We analyzed these factors by comparing users’ compliance with wearing the ring (i.e., their adherence scores) with their sentiments regarding the ring’s ease of use and their personal interest in wearing it after the experiment.

Two surveys were administered to gather users’ impressions on the Oura ring. The first survey was released on the 15th day of the experiment and asked users whether any features were confusing. The second survey that was administered at the end of the experiment on the 31st day asked users if they would like to continue using the ring after the study.

**Seventeen participants reported a willingness to continue using the Oura ring after the experiment.** Out of these seventeen individuals, six respondents had adherence scores above 184, while the rest were in the 160–170 range. Two respondents had relatively low scores of 155 and 130. This indicates that compliance with the usage of a wearable does not automatically equate to affinity to the device: Participants with low or inconsistent adherence to wearing the ring were still interested in continuing its use even after the experiment.

**Figure 5:**
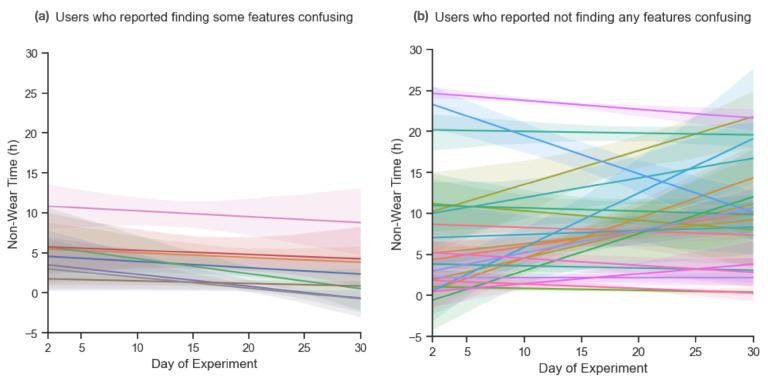
The difference in usage trends between users who reported finding features of the Oura ring confusing and users who did not. (**a**) The non-wear time trends of users who reported some features of the Oura ring confusing. (**b**) The non-wear time trends of users who declared they were not confused by any of Oura’s features.

**Only eight participants reported finding any features confusing.** Responses were mainly focused on uncertainty over how particular behaviors or criteria were measured (e.g., highs and lows of daily movement) as well as confusion over how they were meant to improve themselves using the data provided to them by the ring. Some remarks expressed ambivalence over how the features were intended to be used and how the data were supposed to be read. These feelings of confusion over the ring’s features affected participants from both younger and older age brackets: Three respondents were between the ages of 23 and 28 and five respondents were between the ages of 37 and 49. We might expect these users to have a lower usage, but all of them also had relatively high adherence scores: Five users had scores above 180, with the highest being 193, while the rest did not have scores that went lower than 160. However, these eight users still showed a downwards trend in terms of usage distribution (Figure 5a) as compared to the rest of the cohort who did not report finding any features confusing (Figure 5b). This suggests that feelings of uncertainty over the usage of a wearable device can impact the consistency of a participant’s usage over time but it does not necessarily compromise the participant’s compliance overall.

**Twenty-one out of thirty participants responded to both surveys, four responded to at least one survey, and five did not answer any survey** (Table 3). All in all, each survey had a total of 23 responses. The adherence score did not factor into compliance: Among the five participants who did not participate in either survey, three had an adherence score above 180 and includes the user with the highest score of 195. Likewise, three out of the four participants who only answered one survey had adherence scores above 180.

## 6. Discussion and Conclusions

Our ultimate goal throughout this study was to determine the naturalistic behavior of people when they are given an Oura ring to interact with over a long-term experiment duration. This was so we could confirm the viability of achieving enough participation to gather sufficient data through an in-the-wild setup lasting longer than two weeks. The results confirm that a high participation rate in an in-the-wild study is possible: twenty-six out of thirty-one participants completed the experiment while having contributed enough data for analysis (twenty-four had a participation rate equal to or above 80% and two users had rates that came close at 78%) while only four users had low participation rates that went below 75%.

Other similar in-the-wild studies with wearable technology have achieved similar compliance rates, which further demonstrates the feasibility of experiments with comparable study designs. One study on the Fitbit reported a high user engagement for 73% of the total days of the experiment [12], although it did not account for daily adherence to wearing the device and consistency of usage. In another in-the-wild study that also made use of the Oura Ring to examine the influence of maintaining a sleep tracking routine in one’s daily life, users who were noted to rarely check their Oura application still wore the ring 85% of nights throughout the experiment duration. However, the study also does not factor in the regularity of adherence to wearing the ring in its analysis [24].

When given the opportunity, participants are capable of complying with an experiment format that lasts for several weeks and are able to adhere to a consistent schedule. However, a lack of understanding of the device’s output should be interpreted as having an influence on compliance over time; researchers whose goal is not to examine naturalistic behavior may need to provide their participants with additional training to ensure they understand how to use the ring. It is also important to note that compliance with wearing the device does not necessarily equate to having an affinity to it.

We might expect young people to adhere to the device more than older people. However, older adults in their mid-30s to late 40s still adhere to the use of a novel technology nearly to the same degree as younger adults in their early 20s and 30s. Although gender was found to be a significant contributor to adherence in this particular study, gender still remains a very small factor: Researchers who may conduct similar studies in the future might find that women are slightly more consistent in adhering to wearable technology, but this study does not provide conclusive evidence of this outcome. Overall, we expect that gender does not significantly influence compliance. This tells us that there are no unexpected demographic costs to in-the-wild studies except for recruiting the needed participants: Studies that obtain a broad population are best practice in terms of accessibility and inclusiveness as well as practical outcomes.

It is difficult to ascertain what factors contributed to the insufficient participation rate of some users in the study. Out of the four users with low scores, one did not respond to the survey and the remaining three did not report finding any features confusing. Nevertheless, only one of them declared an interest in wearing the ring outside of the experiment. It is possible that the short engagement of these users is due to a lack of interest in the ring and its features. It could also be as simple as them not having a desire to track their sleep data. Experiment practitioners should take note of this and consider that some individuals may not be motivated to improve themselves and may already be content with their current lifestyle regardless of how efficient they are.

This study does possess several limitations. Participants had to have some English ability in order to join the study, which means that most recruited subjects may have wealthy or upper-middle-class backgrounds. We did not consider socioeconomic status when forming our generalized sample, so there is a possibility that lower-income individuals were underrepresented. Furthermore, our age range, while reasonably diverse, still lacks representation of children, adolescents, late middle-aged people, and elderly people. The compliance of participants may also be attributed to an “authority effect” caused by their formal recruitment through an agency, and we must also consider the potential that compliance may vary across cultures and that the results may only be applicable to the Japanese population. In addition to this, the performance of the PVT may have been a confounding factor. There are also several other confounding factors outside of our ability to measure, such as an individual’s personal taste in fashion and the potential interference of certain jobs to wearing the ring on a regular basis. The surveys that we administered during and after the experiment were not able to inquire into the reasons why certain users had a low participation rate, and there were no specific questions that helped gauge the ring’s exact appeal and level of usability in different contexts, such as at work or during sports. Thus, it is not possible for us to enumerate the potential barriers to adherence. Lastly, as we only tested on one device (the Oura ring), there is a chance that different results may be gathered from the use of another wearable. In our future work, we intend to expand this study further by covering different populations, accounting for children and much older individuals, and asking more questions to gather insight into the potential reasons behind the behavior of users.

## Figures and Tables

**Table 1:** Breakdown of Age Differences Between Men and Women.

Female	Male
23	21
23	22
25	22
28	23
31	24
32	24
33	24
34	25
36	37
37	37
41	40
42	40
43	41
45	45
48	46
49	

**Table 2:** User Participation Rate Based on Adherence Score out of 200.

User No.	Adherence Score	Participation Rate
013	196.99	98%
026	195.34	98%
027	195.57	98%
019	194.12	97%
021	194.62	97%
008	191.18	96%
012	191.06	96%
001	185.64	93%
009	186.94	93%
020	185.62	93%
028	183.31	92%
030	184.95	92%
029	181.30	91%
003	180.66	90%
015	179.19	90%
002	175.87	88%
010	171.55	86%
014	171.32	86%
011	170.06	85%
025	169.99	85%
031	167.98	84%
018	166.63	83%
022	159.14	80%
023	159.98	80%
004	155.70	78%
007	156.59	78%
005	143.95	72%
024	130.39	65%
016	117.16	59%
017	79.51	40%
006	*Dropped*	*Dropped*

**Table 3:** Survey Compliance Rate.

Completion Level	Responses
Responded to Both Surveys	21
Only Answered First Survey	2
Only Answered Second Survey	2
Did Not Answer Any Survey	5

## Data Availability

The data presented in this study can be requested from the corresponding author.
